# Psycho-Somatic Evolution of Patients with Multiple Traumatic Injuries

**DOI:** 10.3390/clinpract14060189

**Published:** 2024-11-11

**Authors:** Mihaela Anghele, Virginia Marina, Cosmina Alina Moscu, Aurelian-Dumitrache Anghele, Liliana Dragomir

**Affiliations:** 1 Clinical-Medical Department, Faculty of Medicine and Pharmacy, “Dunărea de Jos” University, 47 Str. Domnească, 800201 Galati, Romania; mihaela.anghele@ugal.ro (M.A.); lilianadragomir2017@gmail.com (L.D.); 2 Medical Department of Occupational Health, Faculty of Medicine and Pharmacy, “Dunărea de Jos” University, 47 Str. Domnească, 800201 Galati, Romania; 3 Doctoral School, “Dunărea de Jos” University, 800201 Galati, Romania; cosmina_caluian@yahoo.com (C.A.M.); anghele_aurelian@yahoo.com (A.-D.A.)

**Keywords:** posttraumatic stress disorder, multiple traumatic injuries, stress-related growth scale

## Abstract

Background and Objective: In this study, we aimed to identify the factors that could impact the Stress-Related Growth Scale (SRGS) questionnaire administered to patients. Materials and Methods: Participants were asked to complete a written SRGS questionnaire (a translated and approved version in Romania) at varying time intervals relative to the traumatic event. The questionnaire was developed in accordance with legal regulations of the World Health Organization and the European Union for research involving human subjects for medical purposes. It took approximately 15 min to complete. The questionnaire was filled out by the patient or their legal guardian/parent for minors between January 2021 and January 2022. Results: The findings revealed the individual dimensions in the context of the traumatic impact, and the subsequent conclusions could be applied to a larger group with similar traumatic experiences. It is recognized that psychosomatic pathologies can hinder posttraumatic rehabilitation, leading to slower and more challenging recovery. Conclusions: Posttraumatic stress disorder often manifests as chronic development of symptoms characterized by reexperiencing traumatic scenes, avoidance behaviors, negative alterations in cognition, and heightened arousal. Posttraumatic stress disorder (PTSD) is a prevalent, persistent, and psychologically debilitating syndrome that can significantly impair an individual’s ability to cope with life. The etiology and manifestation of this disorder present numerous challenges due to the complexity of defining and diagnosing these conditions. The distribution of men and women affected by posttraumatic stress disorder varies across different sources and cannot be simplified into one explanation. While sex distribution is an important factor, it is not the sole determinant for understanding the various aspects of these disorders. The diversity of stressors has been shown to correlate with changes in SRGS scores, including subtle emotions like shame and guilt, which contribute to the moral injury resulting from trauma.

## 1. Introduction

In recent decades, researchers have discovered that being in the midst of a distressing or traumatic event can lead to various positive life changes.

Many negative life moments can have a powerful influence on a person’s life, and they can occur at various stages from childhood to adulthood and be perceived and processed differently [1]. In the 20th century, there was a growing focus among specialist nurses, psychologists, psychiatrists, neurologists, and social workers on these traumatic events. Research on anxiety disorders has accelerated, leading to the initiation of many multicentric clinical studies that focus on stress and distress in terms that are more clearly defined than before [2].

Posttraumatic development is a phenomenon where individuals experience positive changes after facing extremely negative events [3]. This can lead to better social integration, a deeper understanding of social relationships, increased self-confidence, and the development of effective coping mechanisms. Examples of such negative events include the death of a loved one, a cancer diagnosis for oneself or a close friend or family member, cardiovascular disease, accidents resulting in various forms of disability, and separation from a life partner [2]. 

The survival strategy for these individuals is more eclectic because they combine different learning methods, thus increasing the likelihood of experiencing positive emotions after a negative event. This diversity makes their adaptation more effective in various situations, which sometimes allows for turning challenges into advantages. How people respond to negative events significantly impacts their overall well-being, including their housing, work, relationships, and quality of life. Therefore, scientific research to develop precise tools for dynamically measuring posttraumatic development is crucial for further, more pragmatic studies on these intriguing aspects and for practical applications, particularly in new clinical scenarios.

According to the DSM-5, posttraumatic stress disorder (PTSD) develops when an individual is exposed to a traumatic event involving actual or threatened death, serious injury, or sexual violence. This exposure can occur by directly experiencing the event, witnessing it, learning that a close person experienced it, or repeated exposure to aversive details of traumatic events. PTSD is characterized by symptoms of intrusion, avoidance of trauma-related stimuli, negative alterations in cognition and mood, and heightened arousal and reactivity.

Physical injuries can cause pain and suffering that are challenging to manage, leading to mental anguish and a decreased quality of life. The long-term nature of many multiple trauma injuries can contribute to feelings of hopelessness and powerlessness [4]. Examples include memories of soldiers in wars, earthquakes, diseases, and exams, as various traumatic life events can trigger unpredictable and uncontrollable emotions. This disorder affects not only those who directly experience a disaster but also witnesses, individuals who assist victims, such as emergency medical staff, military personnel, and firefighters, and even friends or family members of those who have undergone trauma.

The COVID-19 pandemic has led to a significant increase in levels of anxiety, depression, and stress among the general population, as well as among patients with acute or chronic medical conditions. Studies show that social isolation, health-related uncertainty, and fear of infection have had a major negative impact on mental health [5,6]. In particular, patients with multiple traumatic injuries may experience heightened anxiety and depression as they face challenging recoveries, and the lack of direct social support can exacerbate feelings of helplessness and loss. Patients may experience a “double impact” of loss, including loss of physical function as well as loss of social interactions and a sense of security, which are essential for complete psychological recovery [7,8]. Furthermore, pandemic-related uncertainties can amplify feelings of vulnerability and may lead to intensified posttraumatic stress among these patients. Also, patients with multiple traumatic injuries who are in the recovery phase may face exacerbated symptoms of posttraumatic stress given the pandemic context. Given that mental state is a significant determinant of physical recovery, it is essential to recognize and address these psychosocial issues in an integrated manner. The pandemic has significantly limited social interactions and access to support networks, which are essential for the psychosomatic recovery of patients with multiple traumatic injuries. The absence of this support can contribute to heightened symptoms of posttraumatic stress and increased feelings of isolation (Sawhney et al., 2020). In a context where traumatized patients are already predisposed to symptoms of depression and anxiety, the lack of emotional support can have long-term consequences for their well-being [9].

## 2. Materials and Methods 

This study included a sample of 187 patients (103 males and 84 females) treated at the Galați Emergency Department who experienced multiple traumatic injuries. To ensure a comprehensive understanding of the participants’ background and reduce generalization, we collected demographic data, including age, sex, residence environment, marital status, smoking habits, and personal medical history related to chronic diseases. Participants’ ages ranged from 19 to 87 years, and they were from both urban and rural areas.

They completed the SRGS questionnaire, and the results and answers were then analyzed in combination with the individual characteristics detected in the group. This research method medically studied the impact of these parameters and their correlations with and implications for psycho-somatic pathologies superposable with post-trauma recovery.

The SRGS questionnaire was translated, and the accepted version in Romania is shown in Appendix A. The questionnaire was completed at a variable time in relation to the traumatic event if they wanted and were able to do so. 

The questionnaire was developed after harmonization with the current rules and laws of the World Health Organization, together with the European Union, in the field of generating studies for humans in medical research projects. The standard questionnaire completion time was approximately 15 min. The questionnaire was filled out by the patient or by the legal guardian/parent of minors between January 2021 and January 2022.

Data collection and processing are fundamental principles of patient anonymity. Approval from the Bioethics Commission of the Galati County Emergency Hospital was obtained to access and collect patients’ personal information.

Patients who were present in the Emergency Ambulance Services in Galați for traumatic events (such as road and car accidents, falls from heights, explosions, violence, and poisoning) were invited to complete the questionnaire after stabilization. Patients had to meet specific inclusion criteria for this research, including having experienced traumatic events in their life history, being treated as patients in the Emergency Department of Galati Hospital, not having any psychiatric pathologies that could influence the validity of the data obtained, and giving consent to be included in the study.

The information was organized to allow for sorting and filtering based on certain types of pathologies and specific patient demographics. Data from our study were analyzed using IBM SPSS Statistics V26 by focusing on contingency tests and descriptive statistical analysis. A 95% confidence interval of variation was allowed in the study of proportions. The Chi-square test (χ2) was used for comparing proportions and testing the sensitivities of the diagnostic methods. This allowed us to highlight associations, relationships, and important interdependencies between variables.

## 3. Results

Our study noted a predominance of urban cases in the group (52 males and 47 females), with the age range spanning from 19 to 87 years and mean age values of 48.95 years (±SD 17.28 years).

There is a nearly equal distribution of tobacco users (43 females and 53 males), married participants (46 females and 48 males), and children (58 girls and 71 boys). The statistics varied between chronic gamblers (31%, OR 2:1) and those with a significant personal pathological history (Table 1).

One-way ANOVA showed no significant differences based on the status variables of participant age, time since the event, or PTSD symptoms associated with the event. However, chi-square analyses revealed significant differences between traumatic event conditions and sex characteristics.

To simplify the statistical analysis and present relevant conclusions, responses from the SRGS questionnaire (Appendix A) will be tracked in a comparative manner by analyzing distributions by gender. The questions will be defined as items (I1, I2, …, I13), numbered according to Table 2, with answers coded in three steps: “strongly disagree”, “somewhat agree”, and “strongly agree”.

Results for 13 items (I.1–I.13) from Table 2

- For I.1, 85 subjects expressed total disagreement (sig 0.065), with 46 of them being women, and 40 agreed, with 26 being men.
- For I.2, related to changes in personality, 48 had positive responses (sig = 0.014), with a higher incidence among 25 males.
- Decision simplification in I.3 was recognized by 50 subjects (sig = 0.017), with the majority being male (35 male patients, OR 2:1).
- For I.4, regarding the absolute value of life, 36 had affirmative responses (sigma = 0.123), with 21 in favor of males.
- Post-trauma adverse consequences in I.5 were acknowledged by 43 patients, 28 of whom were male (sigma = 0.107).
- Post-event insomnia in I.6 was detected in 26 patients (ratio 5:1 in favor of males, sigma = 0.037).
- For I.7, which is about helping others, 32 had positive responses (17 female patients, sigma = 0.533).
- The incidence of self-confidence post-event in I.8 was correlated with male sex (18/2 cases detected among females, sigma = 0.004).
- For I.9, which focused on attention to others, 40 gave affirmative responses, with 24 being male (sigma = 0.722).
- The sincerity of the subject in I.10 had 62 affirmative responses, with an equal distribution by gender (32 female patients), sigma = 0.000.
- The importance of asking for help if needed in I.11 (sigma = 0.190) resulted in 72 positive responses, with only 27 being female patients.
- In I.12, advocacy, although not statistically significantly different (sigma = 0.587), showed an equal distribution between sexes, with 47 female patients and 53 male patients.
- In I.13, feelings of loved ones (family, friends) were validated by 57 patients, with 26 being female patients, sigma = 0.847.

The analysis of the incidence of disorders, such as distrust, insomnia, aggressiveness, and personality changes, was correlated with the distribution of subjects by gender and other factors, such as living in a rural environment, lack of stable employment, substance abuse, and absence of a supportive family environment that actively aids in the patient’s healing process. Additionally, the presence of the pandemic and the restrictions it imposes increase the risk of psycho-affective changes following a traumatic event. 

## 4. Discussion

Posttraumatic stress disorder (PTSD) can develop into a chronic disorder with significant symptoms focused on reexperiencing trauma, specific avoidance behaviors, negative changes in cognition, and heightened arousal. 

PTSD is becoming more prevalent, and it can greatly impact a person’s functioning across various aspects of life. 

Understanding the causes and mechanisms of PTSD requires extensive research and a well-prepared team with proper testing methods. 

In 1980, the DSM-III introduced the crucial diagnosis of PTSD, categorizing 17 symptoms into three groups. After years of debate, the latest version of the Diagnostic and Statistical Manual of Mental Disorders (DSM-5) now recognizes PTSD with 20 symptoms divided into four groups: intrusive thoughts, avoidance behaviors, negative changes in cognition and mood, and significant alterations in arousal and reactivity [10].

The results of our study provide insights into how individuals process trauma and the potential for posttraumatic growth. Specifically, the data indicate that certain factors, such as age, gender, and environmental support, significantly influence individual experiences of growth post-trauma. To build a cohesive narrative, we have expanded on these findings to discuss how the posttraumatic growth observed aligns with the dimensions of resilience, self-perception, and social functioning. Our results underscore that growth does not occur uniformly; rather, it manifests through individual coping mechanisms and external support structures, such as family or community, which are inextricably linked to PTSD recovery processes.

Many papers have explored the criteria for diagnosing PTSD and have learned how to recognize this syndrome by understanding the factors that affect the individuals involved, as well as how new methods can be developed to test for the presence of the syndrome. PTSD is well-known among doctors, and there is an abundant literature in this field. However, clinical studies that use structured interviews are relatively rare, possibly due to ethical implications, patient consent, and the time constraints faced by investigators, who have numerous other responsibilities. As a result, self-assessment scales, such as the “Posttraumatic Diagnostic Scale” (PDS) [11] and the “Event Impact Scale” (EIS) [12], are frequently used in clinical studies. Limitations can arise when focusing solely on PTSD syndrome, as issues affecting the lifestyle and dimensions of life for our subjects may be more complex. There is ongoing debate about the differences in the prevalence of PTSD between male and female subjects. 

Studies in the specialized literature show that working conditions and activities carried out during a certain period of life can have a significant impact on the psyche. These factors can leave lasting consequences, leading some individuals to experience various forms of depression following trauma.

In large cohorts of veterans, the prevalence of PTSD in male and female populations has shown similarities, with statistically higher rates found in men compared to women (13% versus 11%) [13]. Another study found a lower prevalence of PTSD in women compared to men (6.6% versus 5.3%, respectively) [14]. Women are generally less likely to be exposed to combat, but they may develop PTSD following military sexual trauma [15].

In contrast to the civilian population, where 16.7% of those diagnosed with PTSD are 18–29 years old [16], many of the active-duty service members are up to 30 years of age. In nursing facilities for veterans, studies from 2001 to 2005 showed a high risk of PTSD in the age group of 18–24, followed by the 25–29 age group and the 30–29 age group, indicating that PTDS is more common in younger veterans compared to those aged 40 and older [17].

The black population has been diagnosed with PTSD more frequently according to Seal’s conclusion, suggesting a higher prevalence compared to other racial or ethnic groups [18]. Dohrenwend was interested in exploring how PTSD may differ based on race in personal military service [18,19]. The higher prevalence of PTSD in black veterans was linked to increased exposure to war zone stressors, while the development of PTSD in Hispanic veterans was attributed to various factors, such as younger age at enlistment, lower academic skills, and reduced military qualifications.

Between 44 and 72% of veterans associate increased stress levels with their transition back to civilian life. A meta-analytic study including 34 studies found that the severity of PTSD symptoms was significantly correlated with feelings of anger, particularly in military samples [20].

For members of the Canadian Armed Forces, exposure to moral injury during deployments can be an independent risk factor for the development of PTSD over time [21]. 

It has often been observed that high levels of PTSD and emotional problems lead to the occurrence of domestic violence in the families of war veterans. Evans was preoccupied with a survey assessing the influence of PTSD symptoms on family life dynamics [22]. The analysis of Evans and colleagues concluded that family functioning is directly impacted by avoidance symptoms because members struggle to communicate effectively. They also found that hyperarousal symptoms could indirectly affect family functioning by causing family members to focus on minor issues rather that the core values of a healthy family. Reexperiencing symptoms was not found to be significant for family functioning. Recent epidemiological data from the Veterans Health Administration Registers showed that many veteran patients with PTSD have suicidal ideation and subsequent behaviors, which are often linked to depressive mood and other mood disorders [23].

Another important characteristic observed in veterans is the presence of emotional and behavioral disturbances in children [24]. The effects of PTSD on young children are receiving increasing attention from researchers, with only a few studies identifying prevalent factors and causes that reflect responses to early trauma exposure. 

The diverse causes of PTSD are not yet fully understood, and several studies have been conducted. The neuroendocrine system, through neuroendocrine secretions, mediators, and immune systems through cytokines and other immunological factors, is involved in the generation and presence of a vast majority of the symptoms in PTSD [25]. Twenty studies involving a meta-analytical method showed the presence of high levels of proinflammatory cytokines in plasma in a variety of pathologies and biological events. Tumor necrosis factor-alpha promotes inflammation, and interleukin-1beta (IL-1b) and interleukin-6 (IL-6) are prominent in individuals with PTSD compared to healthy controls [26]. A prospective association of C-reactive protein (CRP) and mitogen with the presence of PTSD was formulated because all of these molecules imply more local and general secretion of proinflammatory molecules, putting the organism in a high-arousal state [27]. These findings link to the conclusion that neuroendocrine- and inflammatory-specific modulations are able to act as a preexisting biological basis and risk factor for the development of PTSD due to traumatic events. Increased levels of terminally differentiated T cells together with an altered Th1/Th2 equilibrium may lead to an affected immune status and thus predispose a person to PTSD over various time spans.

Genetic and epigenetic factors play a significant role in many situations, with up to 70% of individual differences contributing to the development of PTSD [28]. DNA methylation, related to specific environmental conditions, can be closely tied to the onset of PTSD in some individuals, along with other contributing factors. Global studies have shown that prolonged or intense exposure to stress can directly impact gene expression in offspring through epigenetic mechanisms, leading to long-term risks associated with PTSD.

The personality of individuals is a predictive factor for the occurrence of stress or burnout syndrome [29].

Various mental illnesses, such as major depressive disorder (MDD), bipolar disorder, and schizophreniform disorders, are often linked to significant changes in subcortical volume. Recently, researchers have explored the connections between morphological changes in subcortical structures and PTSD. In many cases, individuals with PTSD show lower white matter integrity, and these modifications have been examined in their brains [30]. Logue conducted a neuroimaging study on the PTSD spectrum by comparing eight subcortical structural volumes between PTSD patients and healthy individuals [31]. It was found that a smaller hippocampal volume was specifically linked to PTSD, while a smaller amygdala volume did not show a significant correlation. 

Another study has shown that the increase in burnout among individuals is directly proportional to the nature of their work [32].

There are three types of approaches to preventing the onset of PTSD: primary prevention (aimed at preventing exposure to the traumatic event itself), secondary prevention (addressing PTSD symptoms after a traumatic event has occurred), and tertiary prevention (intervening after PTSD symptoms have been identified in a patient).

Pharmacological studies have sought to determine the impact of stress on memory formation mechanisms. These studies have explored drugs that can influence the hypothalamic–pituitary–adrenal (HPA) axis, drugs that affect the autonomic nervous system (especially the sympathetic nervous system), and other medications, such as opioids. Evidence indicates that pharmacological prevention programs are most effective when implemented before or shortly after a traumatic event. Sympatholytic drugs, specifically alpha and beta blockers, have been shown to be more effective in the primary prevention of PTSD [33]. 

Many guidelines recommend trauma-focused psychological interventions as first-line treatments for PTSD. These interventions include cognitive behavioral therapy, cognitive processing therapy, cognitive therapy, cognitive restructuring, and coping skills therapy. Exposure-based therapies, eye movement desensitization and reprocessing, hypnosis hypnotherapy, and brief eclectic psychotherapy have also shown significant benefits in treating PTSD symptoms [34]. Jonas et al. conducted a systematic review and network meta-analysis of PTSD management studies suggesting that all psychological treatments are effective in alleviating PTSD symptoms, with exposure-based treatments being particularly efficient [35]. Kline conducted a meta-analysis of the long-term effects of in-person psychotherapy for PTSD in 32 randomized controlled trials involving 2935 patients over a six-month period [36].

The research shows that all treatments led to significant improvements in individual outcomes, with exposure therapies demonstrating particularly therapeutic effects compared to other treatments [37]. The prognosis largely depends on the presentation time to the doctor and the patient’s comorbidities [38].

Pharmacological interventions for PTSD often involve antidepressants, such as selective serotonin reuptake inhibitors, serotonin and norepinephrine reuptake inhibitors, monoamine oxidase inhibitors, as well as sympatholytic drugs, like alpha-blockers, antipsychotics, anticonvulsants, and benzodiazepines. Drugs like fluoxetine, paroxetine, sertraline, and topiramate are commonly used in the treatment of PTSD. Risperidone and venlafaxine have also been shown to be effective treatment options. In a meta-analysis of 28 studies involving 4817 subjects, paroxetine and topiramate were found to be more effective than many other drugs in alleviating PTSD symptoms [35]. 

The main goals of pharmacotherapy are to reduce morbidity and prevent complications [39].

The long-term prognosis for individuals with PTSD is influenced by their ability to cope with stress, substance abuse issues, and the presence of supportive social network.

It is crucial for individuals to stick to a personalized treatment plan. Research suggests that around 30% of individuals may fully recover, while another 40% may see improvement with treatment, though some mild symptoms may persist. 

Variability in psychiatric symptoms among patients with multiple traumatic injuries is a significant consideration in this study. The symptoms of psychiatric conditions, such as posttraumatic stress disorder (PTSD), anxiety, and depression, can indeed vary widely between individuals. This variability can be attributed to several factors. Age, gender, and socio-economic status can influence how individuals process trauma and, subsequently, whether they develop psychiatric symptoms [40,41]. The nature and severity of the traumatic injury can also affect psychiatric outcomes. Individuals with a prior history of psychiatric disorders may have an increased risk of exacerbated symptoms following trauma. Conversely, those without such a history might exhibit resilience or less pronounced symptoms [1]. We analyzed the vulnerabilities of the subjects and identified personality traits and gender and age differences [42]. Patients who experience more severe or life-threatening injuries may be more likely to develop intense PTSD symptoms, while those with less severe injuries might exhibit milder forms of anxiety or stress responses [43]. However, patients with strong support networks often exhibit better coping mechanisms, while those with limited support may experience more pronounced psychiatric symptoms [12].

The results of this study contribute to a deeper understanding of the psychosomatic evolution of patients with multiple traumatic injuries. The findings suggest that traumatic experiences can significantly impact both mental health and physical recovery, especially in a post-pandemic context. This study supports the idea that trauma can lead to complex psychosomatic responses and the need for a holistic approach in post-trauma care considering both psychological and physical aspects. This study highlights that exploring the impact of specific types of trauma and their relationship to various demographic factors, such as age, gender, and social support, could enhance our understanding of recovery dynamics.

Our discussion extends beyond summarizing the results to including an in-depth analysis of how posttraumatic growth is influenced by specific life changes, such as shifts in self-confidence or interpersonal relationships. This interpretation allows us to contextualize our findings within the broader trauma literature by connecting individual questionnaire responses to established posttraumatic growth frameworks. By positioning our findings within this theoretical and empirical context, we aim to bridge any perceived gaps between data presentation and the narrative discussion, thus offering a clearer picture of the real-world implications of our study.

### Study Limitations

This study has several limitations that should be acknowledged. First, the data were collected from a single hospital setting, which may limit the generalizability of the findings to other healthcare environments or populations. The specific characteristics of the sample, including demographic factors, such as urban versus rural residence, may also influence the applicability of the results to broader patient groups. Additionally, the retrospective nature of the study and the reliance on self-reported data introduce the possibility of recall bias, as participants may have varying degrees of accuracy in recalling their experiences. 

One limitation of this study is the delay in the publication process, which was primarily due to disruptions caused by the COVID-19 pandemic. Data collection and subsequent analysis were extended over nearly three years, which may affect the immediacy and relevance of the findings. As a result, certain findings should be interpreted with caution, as they may not fully reflect recent developments in posttraumatic stress disorder research and patient recovery dynamics. This delay underscores the need for ongoing studies to validate and expand upon these results within current contexts.

## 5. Conclusions

The discrepancy in the prevalence of posttraumatic stress disorder between men and women remains controversial. Research has shown that sex distribution is an important marker for defining statistically significant differences. Different stressor subtypes, such as feelings of shame and guilt, have been found to correlate with assault.

This study showed positive outcomes for psychological prevention measures, with training and repetitiveness being components with psychoeducational potential. The behavior of patients who try to hide their conditions can provide an important lesson for clinical practice [9].

Equally important is the need for a skills-based component that can address stress responses. This includes various anxiety reduction and relaxation techniques, coping strategies, identifying thoughts, managing emotions and regulating bodily tensions, choice of action, attention, control, and regulation of emotions. Addressing these issues actively reduces posttraumatic phenomena in patients.

Since electric scooters were launched in 2017, they have become increasingly popular among adults, adolescents, and children. However, it is very important to note that while electric-scooter-related head trauma is often mild, it should not be taken lightly, as it can lead to long-term consequences, including posttraumatic stress disorder [44].

## Figures and Tables

**Table 1:** The significance of the variability of personal characteristics on the psycho-somatic evolution of the studied group of patients.

Variability	Characteristic	Gender		Chi-Square Test (Sigma)	Gender					
		Female	Male		Female			Male		
		Number	Number		Environment			Environment		
					Urban	Rural	Chi-Square Test (Sigma)	Urban	Rural	Chi-Square Test (Sigma)
					Number	Number		Number	Number	
Environment	Urban	47	52		47	0		52	0	
	Rural	37	51		0	37		0	51	
Smokers	Yes	43	53	0.971	24	19	0.979	27	26	0.924
	No	41	50		23	18		25	25	
Married	Yes	46	48	0.267	30	16	0.06	28	20	0.137
	No	38	55		17	21		24	31	
Chronic disease carrier	No	58	71	0.986	33	25	0.795	39	32	0.179
	Yes	26	32		14	12		13	19	
Children	No	36	45	0.909	21	15	0.703	23	22	0.911
	Yes	48	58		26	22		29	29	
Related pathologies	No	64	60	0.010	36	28	0.922	29	31	0.606
	Yes	20	43		11	9		23	20	
Job	No occupation	16	11	0.069	9	7	0.966	4	7	0.643
	Employed	32	41		18	14		20	21	
	Unemployed	19	14		11	8		9	5	
	Retired	12	29		7	5		14	15	
	Student	5	8		2	3		5	3	

**Table 2:** Results of SRGS (Stress-Related Growth Scale Questionnaire).

Items	Answers	Gender														
		Female		Male		Chi Square	Female					Male				
		Count	Row N%	Count	Row N %		Environment				Chi Square	Environment				Chi Square
							Urban		Rural			Urban		Rural		
							Count	Row N%	Count	Row N%		Count	Row N%	Count	Row N%	
I.1. Did you experience trauma, or a particular event in the year of disease onset? Such as: accidents, loss of a loved one, job loss, divorce etc.?	Strongly disagree	46	54.1%	39	45.9%	0.065	28	60.9%	18	39.1%	0.2425	20	51.3%	19	48.7%	0.8715
	Somewhat agree	24	38.7%	38	61.3%		10	41.7%	14	58.3%		18	47.4%	20	52.6%	
	Strongly agree	14	35%	26	65%		9	64.3%	5	35.7%		14	53.8%	12	46.2%	
I.2. After the episode did you notice any changes in your personality traits? That is, have you become friendlier or angrier with others?	Strongly disagree	48	52.7%	43	47.3%	0.014	28	58.3%	20	41.7%	0.288	23	53.5%	20	46.5%	0.483
	Somewhat agree	13	27.1%	35	72.9%		9	69.2%	4	30.8%		19	54.3%	16	45.7%	
	Strongly agree	23	47.9%	25	52.1%		10	43.5%	13	56.5%		10	40%	15	60%	
I.3. Do you find that after that episode you made decisions easier?	Strongly disagree	54	54%	46	46%	0.017	35	64.8%	19	35.2%	0.09	22	47.8%	24	52.2%	0.846
	Somewhat agree	15	40.5%	22	59.5%		6	40%	9	60%		11	50%	11	50%	
	Strongly agree	15	30%	35	70%		5	40%	9	60%		19	54.3%	16	45.7%	
I.4. Does your life seem more or less valuable after that loss/change?	Strongly disagree	48	52.2%	44	47.8%	0.123	28	58.3%	20	41.7%	0.878	21	47.7%	23	52.3%	0.228
	Somewhat agree	21	35.6%	38	64.4%		11	52.4%	10	47.6%		23	60.5%	15	39.5%	
	Strongly agree	15	41.7%	21	58.3%		8	53.3%	7	46.7%		8	38.1%	13	61.9%	
I.5. Have you been able to resolve the problems that arose later? For example, were you are able to get a job if the trauma was a loss of your job?	Strongly disagree	52	52%	48	48%	0.107	32	61.5%	20	38.5%	0.321	24	50%	24	50%	0.333
	Somewhat agree	17	38.6%	27	61.4%		9	52.9%	8	47.1%		11	40.7%	16	59.3%	
	Strongly agree	15	34.9%	28	65.1%		6	40%	9	60%		17	60.7%	11	39.3%	
I.6. After that episode did you suffer from insomnia?	Strongly disagree	53	51%	51	49%	0.037 *	31	58.5%	22	41.5%	0.589	28	54.9%	23	45.1%	0.527
	Somewhat agree	25	43.9%	32	56.1%		12	48%	13	52%		16	50%	16	50%	
	Strongly agree	6	23.1%	20	76.9%		4	66.7%	2	33.3%		8	40%	12	60%	
I.7. Do you now help those around you more?	Strongly disagree	45	44.6%	56	55.4%	0.533	29	64.4%	16	35.6%	0.241	24	42.9%	32	57.1%	0.192
	Somewhat agree	22	40.7%	32	59.3%		10	45.5%	12	54.5%		18	56.3%	14	43.8%	
	Strongly agree	17	53.1%	15	46.9%		8	47.1%	9	52.9%		10	66.7%	5	33.3%	
I.8. Do you have more confidence in yourself now than you did before that trauma?	Strongly disagree	60	49.2%	62	50.8%	0.004 *	36	60%	24	40%	0.494	32	51.6%	30	48.4%	0.208
	Somewhat agree	22	48.9%	23	51.1%		10	45.5%	12	54.5%		14	60.9%	9	39.1%	
	Strongly agree	2	10%	18	90%		1	50%	1	50%		6	33.3%	12	66.7%	
I.9. Are you really attentive when people talk to you about their problems? Or do you consider that everyone has to solve their own problems?	Strongly disagree	51	47.2%	57	52.8%	0.722	30	58.8%	21	41.8%	0.793	24	42.1%	33	57.9%	0.106
	Somewhat agree	17	43.6%	22	56.4%		9	52.9%	8	47.1%		15	68.2%	7	31.8%	
	Strongly agree	16	40%	24	60%		8	50%	8	50%		13	54.2%	11	45.8%	
I.10. Are you more honest now with the people around you?	Strongly disagree	46	52.9%	41	47.1%	0.000	28	60.9%	18	39.1%	0.406	21	51.2%	20	48.8%	0.039 *
	Somewhat agree	6	15.8%	32	84.2%		4	66.7%	2	33.3%		11	34.4%	21	65.6%	
	Strongly agree	32	51.6%	30	48.4%		15	46.9%	17	53.1%		20	66.7%	10	33.3%	
I.11. Is it normal to ask for help when you need it?	Strongly disagree	32	53.3%	28	46.7%	0.190	22	68.8%	10	32.3%	0.014 *	11	39.3%	17	60.7%	0.287
	Somewhat agree	25	45.5%	30	54.5%		8	32%	17	68%		18	60%	12	40%	
	Strongly agree	27	37.5%	45	62.5%		17	63%	10	37%		23	51.1%	22	48.9%	
I.12. Is it normal to defend your rights?	Strongly disagree	5	55.6%	4	44.4%	0.587	2	40%	3	60%	0.647	3	75%	1	25%	0.578
	Somewhat agree	32	41%	46	59%		17	53.1%	15	46.9%		22	47.8%	24	62.2%	
	Strongly agree	47	47%	53	53%		28	59.6%	19	40.4%		27	50.9%	26	49.1%	
I.13. Do you think your family really cares about you? What about your friends?	Strongly disagree	19	41.3%	27	58.7%	0.847	10	52.6%	9	47.4%	0.871	13	48.1%	14	51.9%	0.596
	Somewhat agree	39	46.4%	45	53.6%		23	59%	16	41%		21	46.7%	24	53.3%	
	Strongly agree	26	45.6%	31	54.4%		14	53.8%	12	46.2%		18	58.1%	13	41.9%	

## Data Availability

The dataset is available on request from the authors.

## Abbreviations

DNA	Acid deoxyribonucleic
CRP	C-reactive protein
DSM	Diagnostic and Statistical Manual
DSM III	Diagnostic and Statistical Manual (third edition)
END	Dysfunctional negative emotions
ENF	Functional negative emotions
HPA	Hypothalamic–pituitary–adrenal
MDD	Major depressive disorder
PDA	Profile of affective dysfunctions
PDS	Posttraumatic Diagnostic Scale
SRGS	Stress-Related Growth Scale
PTSD	Posttraumatic stress disorder
SD	Standard deviation
STD	Total distress score

## Appendix A. SRGS Questionnaire

I.1. Did you experience trauma, or a particular event in the year of disease onset? Such as: accidents, loss of a loved one, job loss, divorce etc.?
I.2. After the episode did you notice any changes in your personality traits? That is, have you become friendlier or angrier with others?
I.3. Do you find that after that episode you made decisions easier?
I.4. Does your life seem more or less valuable after that loss/change?
I.5. Have you been able to resolve the problems that arose later? For example, were you are able to get a job if the trauma was a loss of your job?
I.6. After that episode did you suffer from insomnia?
I.7. Do you now help those around you more?
I.8. Do you have more confidence in yourself now than you did before that trauma?
I.9. Are you really attentive when people talk to you about their problems? Or do you consider that everyone has to solve their own problems?
I.10. Are you more honest now with the people around you?
I.11. Is it normal to ask for help when you need it?
I.12. Is it normal to defend your rights?
I.13. Do you think your family really cares about you? What about your friends?
